# A Flow Induced Autoimmune Response and Accelerated Senescence of Red Blood Cells in Cardiovascular Devices

**DOI:** 10.1038/s41598-019-55924-y

**Published:** 2019-12-19

**Authors:** James P. Buerck, Dustin K. Burke, David W. Schmidtke, Trevor A. Snyder, Dimitrios Papavassiliou, Edgar A. O’Rear

**Affiliations:** 1 School of Chemical, Biological and Materials Engineering, University of Oklahoma, Norman, OK 73019 USA; 2 Institute for Biomedical Engineering, Science and Technology, University of Oklahoma, Norman, OK 73019 USA; 3 Department of Bioengineering, University of Texas at Dallas, 800 W. Campbell Rd., Richardson, TX 75083 USA; 4 VADovations, 1333 Cornell Parkway, Oklahoma City, OK 73108 USA; 5 Present Address: CorWave, SA, 92110 Clichy, France

**Keywords:** Cardiovascular diseases, Cardiac device therapy, Biomedical engineering

## Abstract

Red blood cells (RBCs) passing through heart pumps, prosthetic heart valves and other cardiovascular devices undergo early senescence attributed to non-physiologic forces. We hypothesized that mechanical trauma accelerates aging by deformation of membrane proteins to cause binding of naturally occurring IgG. RBCs isolated from blood of healthy volunteers were exposed to high shear stress in a viscometer or microfluidics channel to mimic mechanical trauma and then incubated with autologous plasma. Increased binding of IgG was observed indicating forces caused conformational changes in a membrane protein exposing an epitope(s), probably the senescent cell antigen of band 3. The binding of immunoglobulin suggests it plays a role in the premature sequestration and phagocytosis of RBCs in the spleen. Measurement of IgG holds promise as a marker foreshadowing complications in cardiovascular patients and as a means to improve the design of medical devices in which RBCs are susceptible to sublethal trauma.

## Introduction

Flow represents an important stimulus of mechanosensing for many fundamental biological processes. That is particularly true for certain functions of the circulatory system. The action of shearing blood flow on vessel walls causes the release of nitric oxide (NO), drives formation of catch bonds during leukocyte rolling, stimulates expression of fibrinolytic trigger tPA, and results in reduced presentation of inflammatory molecules^1–6^. In developmental biology, flow affects gene expression and cell differentiation setting them, for example, on a path to formation of distinct heart chambers^7^ and may play an additional role in development by determining arterial or venous cell identity^8^. Through such mechanisms, the physical action of shear stress triggers biochemical responses important to functioning and development of the circulatory system.

Shear stress is also important in the initiation and progression of certain pathologies. In particular, flow has been integral to understanding of atherosclerosis and the localization of plaques. Disturbed flow promotes increased expression of pro-inflammatory species like E-selectin and VCAM-1^9,10^. Mechanical trauma, a pathology distinctively attributable to flow, occurs when components of blood encounter non-physiologic forces during extracorporeal circulation with consequences for platelets^11,12^, white cells^13,14^ and von Willebrand Factor, vWF^15,16^ in addition to erythrocytes(RBCs).

Once a major concern in mechanical trauma, hemolysis has become less of an issue with design improvements of prosthetic heart valves and heart pumps. A less apparent manifestation of harm is reduced ability of injured RBCs to survive the microcirculation. As early as 1962, sublethal damage was evident in animal studies of extracorporeal circulation by shortened circulatory half-lives and anemia^17^. While flow in contemporary prosthetic heart valves causes little or no hemolysis, the stresses present do reduce cell lifespans by approximately 20%^18^. In similar fashion, the high shear environment in ventricular assist devices (VADs) has been linked to markedly abridged circulatory lifespans for RBCs^19^. This is important because accelerated removal contributes to anemia for an individual and/or means an added metabolic load to replace the lost cells. Loss of RBCs has been attributed to reduced deformability and early capture in the spleen^20^. *In vitro* experiments by Velker^21^ in 1977 first established stiffness of RBCs after shear exposure, a result later confirmed by a number of other groups^22–26^.

Sublethal mechanical trauma is in fact known to cause a shortened mean circulatory lifespan for red blood cells. Nanjappa^27^ found that the half-life of re-infused Cr^51^-labelled RBCs in the dog decreased with the length of exposure time by 22–60% after low shear stress *ex vivo* (~9 Pa). This research observation fits with clinical findings for circulatory lives of RBCs from prosthetic heart valve patients and ventricular assist devices. Compared to controls (122 ± 23 days), patients with biologic heart valves (103 ± 15 days) and mechanical valves (98.8 ± 23 days) have shorter mean RBC lifespans^28^. Likewise, mean RBC lifespans for patients on continuous flow left ventricular assist devices have been reported to be as low as 30 days^19^. This premature elimination of cells after blood trauma indicates more subtle, sublethal forms of damage may be involved and hints at underlying mechanisms similar to those effecting removal of the senescent RBC.

Increased rigidity after non-physiological shear is a characteristic shared with senescent red blood cells that contributes to their routine removal after a normal 120 day lifespan^20,29^. Years ago Kameneva recognized the similarity between cells naturally aged and those exposed to mechanical stress^30^. To our knowledge though, no group has explored links between shear stress and other widely held theories related to senescence. We considered that mechanisms for physiologic elimination of old RBCs might offer insights into the pathology of mechanical trauma.

Rubin has suggested that the two main models of RBC aging are eryptosis, a variation of apoptosis, and band 3 clustering^31^. These theories tend to focus on biochemical aspects that may act in concert with altered deformability^32^. That is, stiff cells will move through the spleen more slowly, affording greater opportunity for recognition of opsonins by macrophages and phagocytosis. One theory for aging involves attachment of methemoglobin to the interior surface of the RBC membrane or other stimulus to promote aggregation of the integral structural transmembrane protein band 3 (SLC4A1, a solute carrier family 4, anion exchanger, member 1)^33,34^; according to this theory, subsequent binding of naturally occurring antibodies(NAbs) to these band 3 clusters promotes capture of the cell in the spleen^35^.

Band 3 contains a senescent antigen, identified by Kay, that has epitopes for NAbs^36,37^. Since the senescent antigen is present on band 3 in young cells^38^, a key step during aging must somehow bring about greater availability. That may occur by aggregation, enzymatic modification or molecular rearrangement. It is conceivable that deformation of a young cell due to flow will induce accessibility. For example, conformational changes in membrane proteins might expose the senescent antigen^39^ as a result of shear. Moreover, it seems plausible that enhanced mobility of membrane proteins during shear could facilitate band 3 clustering and conformational change. By attaching latex beads to the RBC membrane and observing single cells during “tank tread” flow in the rheoscope, Fischer demonstrated relative motion within the surface of the membrane^40^. Such motion should increase the interaction of molecules of band 3 with greater prospects for aggregation and NAbs binding.

We have shown that binding of autologous immunoglobulin G(IgG) to RBCs occurs at exposure times and stress levels found in cardiovascular devices and conclude that it may be a contributing factor to premature removal of red blood cells in patients with these devices. Externalization of phosphatidylserine (PS) on the outer surface of the cell and eryptosis, another prospective basis for removal of senescent RBCs^39,41,42^, was not found in the present study to be a significant factor.

## Results

### IgG binding to RBC membrane as a result of varying exposure time/shear stress to washed RBC

**Figure 1:**
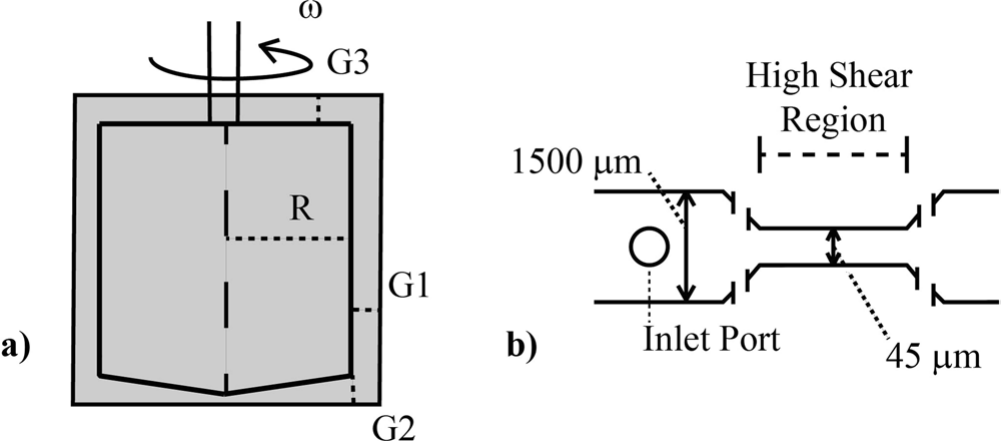
Two methods of shear were used for varying testing conditions. (**a**) The effect of stress magnitude was examined with a viscometer designed for uniform shear with G_1_ = G_2_, G_3_ was machined to give a volume average shear equal to that in the concentric cylinder and cone-and-plate sections. (**b**) The high shear region in the microfluidic channels were varied in length, from 0.7 to 11.6 mm, to vary time of shear to RBCs at a shear rate of 100,000 s^−1^. A control for the microfluidic devices with no high shear region was also used.

**Figure 2:**
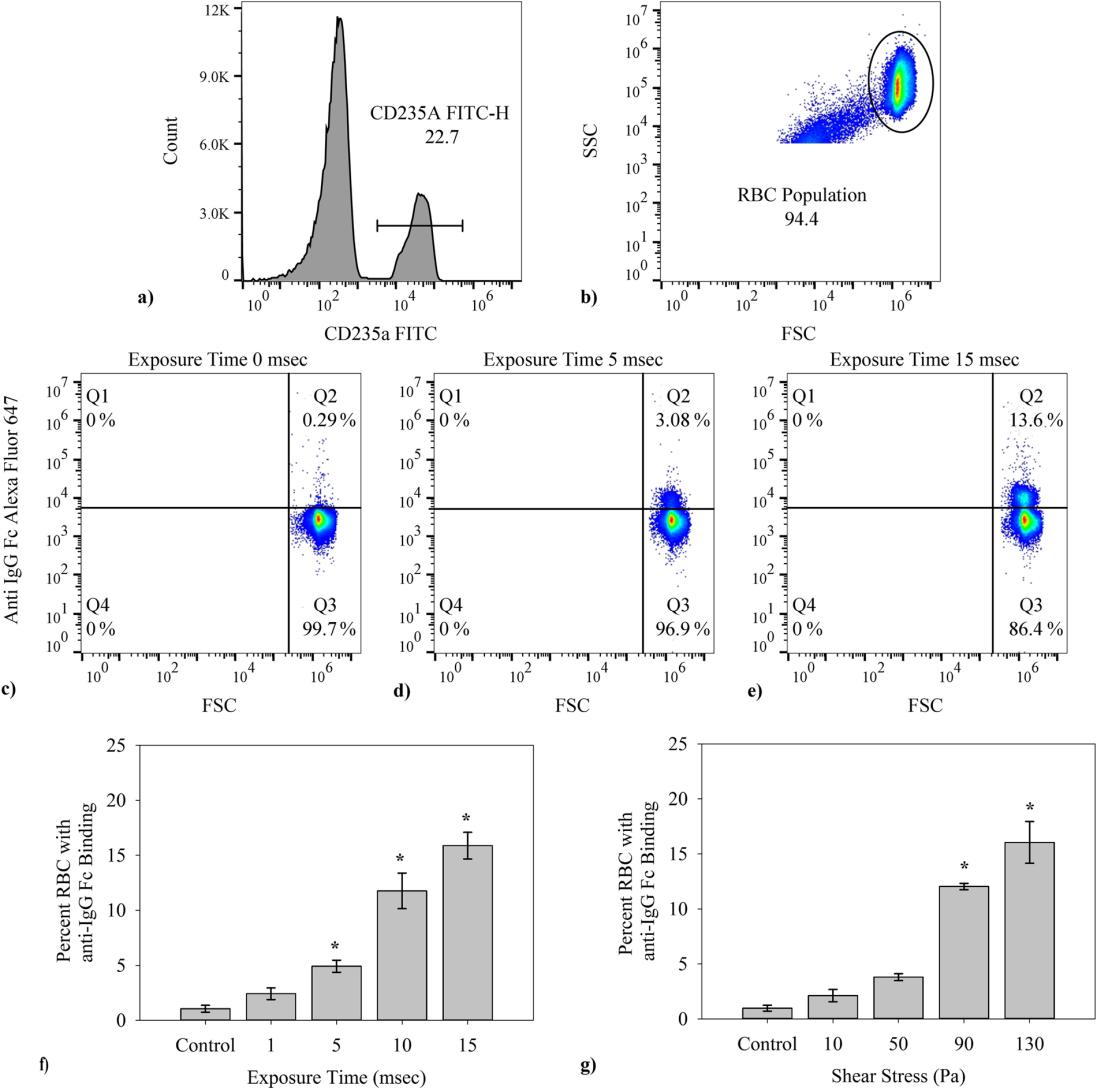
The general flow cytometry methods used in determining percent of cells which fluoresced are shown. Flow cytometry results were collected via the BD Accuri C6 flow cytometer then further analyzed using FlowJo software. (**a**) In every case the events were first gated for presence of CD235a (FITC conjugated), (**b**) then additionally gated according to FSC-SSC to exclude any cellular debris or microparticles. (**c**–**e**) Representative output from the BD Accuri C6 Flow Cytometer for RBCs in a control, 5 msec and 15 msec high shear exposure time samples, respectively from left to right. Each y-axis corresponds to fluorescence intensity from the FL4-anti-IgG Alexa Fluor 647 conjugate and the x-axis shows FSC, relating to event size. Each individual test was run for 75,000 total RBCs. (**f**) The percent of CD235a + RBCs with IgG attached grew with duration of shear after a single exposure in a microfluidics channel at 100,000 s^−1^ (n = 5, p < 0.001). (**g**) The fraction of CD235a + RBCs binding IgG increased with the magnitude of the stress in the viscometer for 2 min. (n = 5, p < 0.001) Data presented in (**f**,**g**) is the average ± standard error, with * representing significance according to post hoc Tukey HSD. Additional tests for Alexa Fluor 647 Mouse IgG2a, k Isotype Ctrl Antibody to account for the possibility of non-specific binding was omitted in (**f**,**g**), having on average 0 ± 0.1% average binding across all tests.

**Figure 3:**
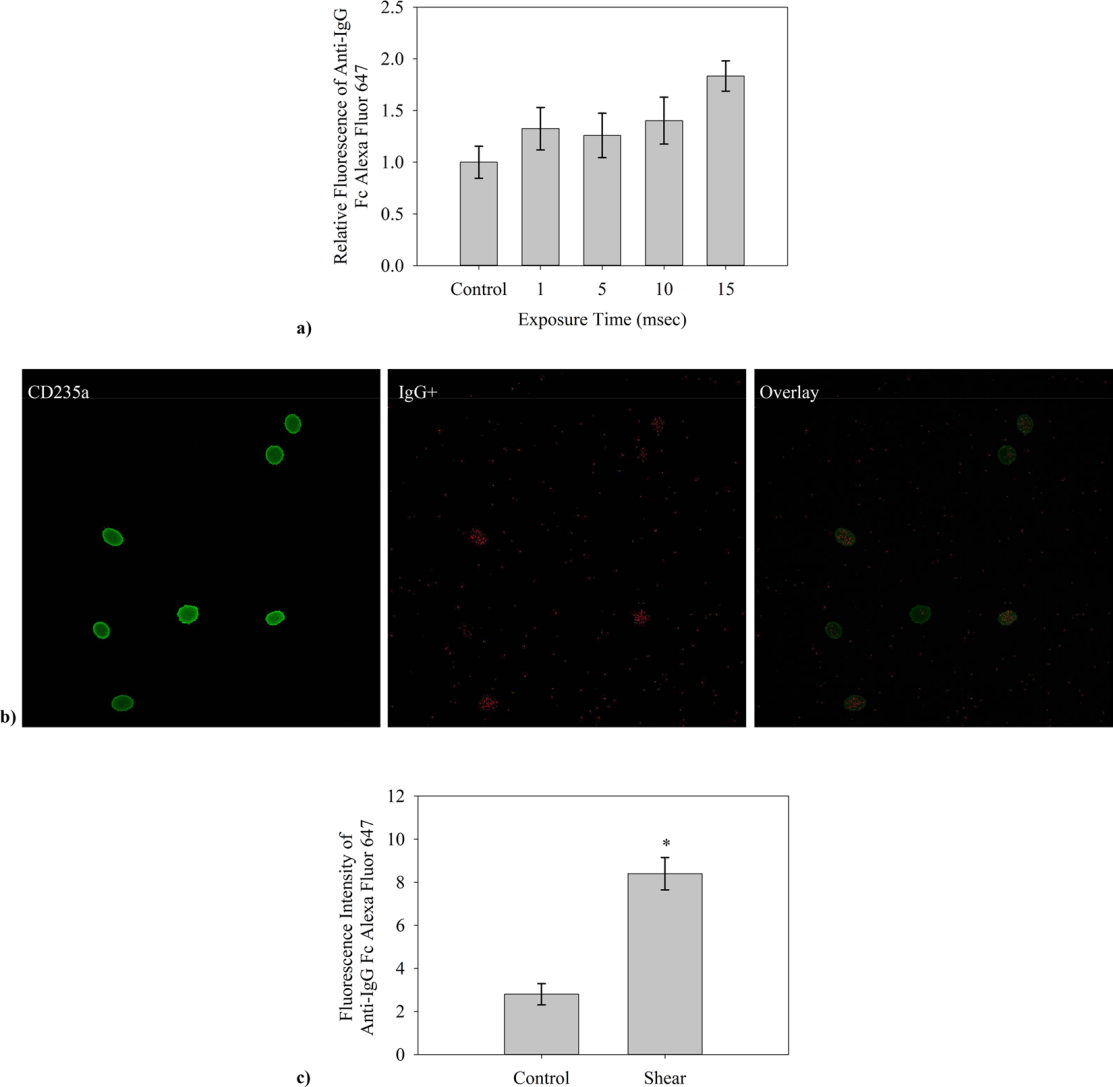
An average increase in fluorescence of RBCs which presented anti-IgG Fc Alexa Fluor 647 fluorescence was presented by data collected via flow cytometry and in collected confocal microscopy images of live cells in media. (**a**) The average fluorescence of the control flow cytometry results was found. That value was then taken as a reference for the relative increase in fluorescence recorded by flow cytometry(n = 5). (**b**) Confocal microscopy images shown are from left to right accordingly, anti-CD235a fluorescence, anti-IgG Fc fluorescence, and an overlay of the two images to show binding presence across the RBC. (**c**) Confocal microscope images were further analyzed by measuring the average intensity above background noise. Fluorescent intensity is presented as mean fluorescence above the background of the image.

Nonphysiologic levels of shear produced *in vitro*, through use of two shear methods (a microfluidics channel and a viscometer, Fig. 1, described in Materials and Methods), cause autologous IgG to bind to RBCs. Typical findings from flow cytometry (Fig. 2) indicate a significant increase in IgG-positive RBCs after flow through the microfluidics channel and subsequent incubation with autologous plasma. Positive events in the upper right quadrant represent RBCs with fluorescently tagged antibody to human IgG. For the specific set of samples in Fig. 2(c–e) with exposure times to high shear of 0 (control low shear channel), 5 and 15 msec respectively at a wall shear rate of 100,000 s^−1^, the percent of cells binding are respectively 0.3%, 3.1% and 13.6%, after excluding microparticles and cellular debris by gating specifically for events of size (forward scatter, FSC) and side scatter (SSC) within the range for RBCs. The fraction of cells binding IgG rises steadily in a statistically significant way (p < 0.001) as the exposure time is increased (Fig. 2f) as well as the magnitude of shear stress itself (Fig. 2g, see below). Average percentage of cells with antibody increases from a control value of 1.0% up to a value of 15.9% at the maximum exposure time tested, across the combined average of all individual tests. This control represents testing of washed RBCs, not run through a channel. Values for the straight channel lacking a high shear region are similar at 1.6 ± 0.5%. Flow cytometry results are also suggestive (p = 0.07) of greater damage to IgG-positive cells with higher average fluorescence occurring as exposure time increased (Fig. 3a). Confocal microscopy(Fig. 3b) of cells incubated with the same probes for CD235a and IgG confirmed the presence of immunoglobulin on RBCs after shear. 50 cells in each experiment(n = 3), control and shear samples (10 msec exposure to 100,000 s^−1^), were analyzed using FIJI ImageJ software and RBCs with detectable fluorescence above the background were recorded. Analysis of fluorescence in confocal microscopy also supported flow cytometry results, yielding a higher average fluorescence intensity of positive cells recorded after 10 msec shear at 100,000 s^−1^ (Fig. 3c). Fluorescence intensity of anti-IgG Fc Alexa Fluor 647 positive sheared cells is much higher than that for anti-IgG positive control cells, approximately 3 times greater according to confocal microscopy images.

In general, the effects of mechanical trauma on blood cells have been found to depend on both shear stress and exposure time. We carried out experiments with a viscometer to investigate immunoglobulin attachment as a function of shear stress over the range of 0–130 Pa. Binding of IgG also depends on magnitude of shear stress(Fig. 2g). The results for unsheared control sample shows on average 1.1% cells testing positive for IgG, similar to the value for the zero shear control in the exposure time experiments. Extent of binding increases with shear stress starting at 0 Pa with a value of 1.1% for a 2 min exposure and rising to 16.0% at 130 Pa.

### Phosphatidylserine exposure on RBCs after exposure to shear stress

**Figure 4:**
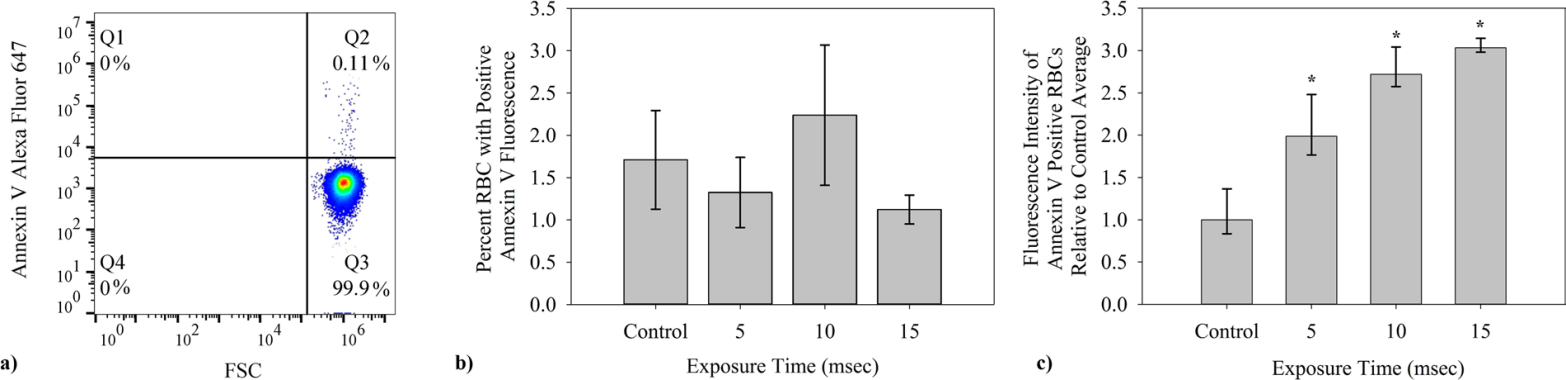
Average percent of cells which bind Annexin V Alexa Fluor 647 was also studied after shear stress in microfluidic channels. (**a**) Similar to the FC detection of IgG presence, presence of PS on RBCs was determined using first gates for CD235a FITC fluorescence and on event size by FSC-SSC. (**b**) The fraction of red blood cells with externalized PS as determined by binding of fluorescently labeled Annexin V was small and did not increase with duration of shear (n = 5). (**c**) The average fluorescence of the control flow cytometry results was found. That value was then taken as a reference for the relative increase in fluorescence recorded by flow cytometry. The amount of PS fluorescence in the positive subpopulation did increase significantly with exposure time(n = 5, p = 0.01).

For the red blood cell, externalized PS marks the cell for eryptosis^41^. Eryptosis potentially offers an alternative mechanism to explain elimination of mechanically traumatized cells. Fluorescently labelled Annexin V was used with flow cytometry to investigate the presence of externalized PS on cells and microparticles (Fig. 4a).

Only a small fraction of cells(1–3%) bound Annexin V and the concentration does not increase over the control level with exposure time(Fig. 4b). While the number of RBCs expressing PS does not increase, those that do are susceptible to greater exposure of PS with shear over the range of 0–15 msec(Fig. 4c). Results shown are the fluorescence increase, relative to the control average. Cells which expressed Annexin V fluorescence had on average 3.0 times greater FL4 (Alexa Fluor 647) values after 15 msec exposure in microfluidic channels than the control.

## Discussion

**Figure 5:**
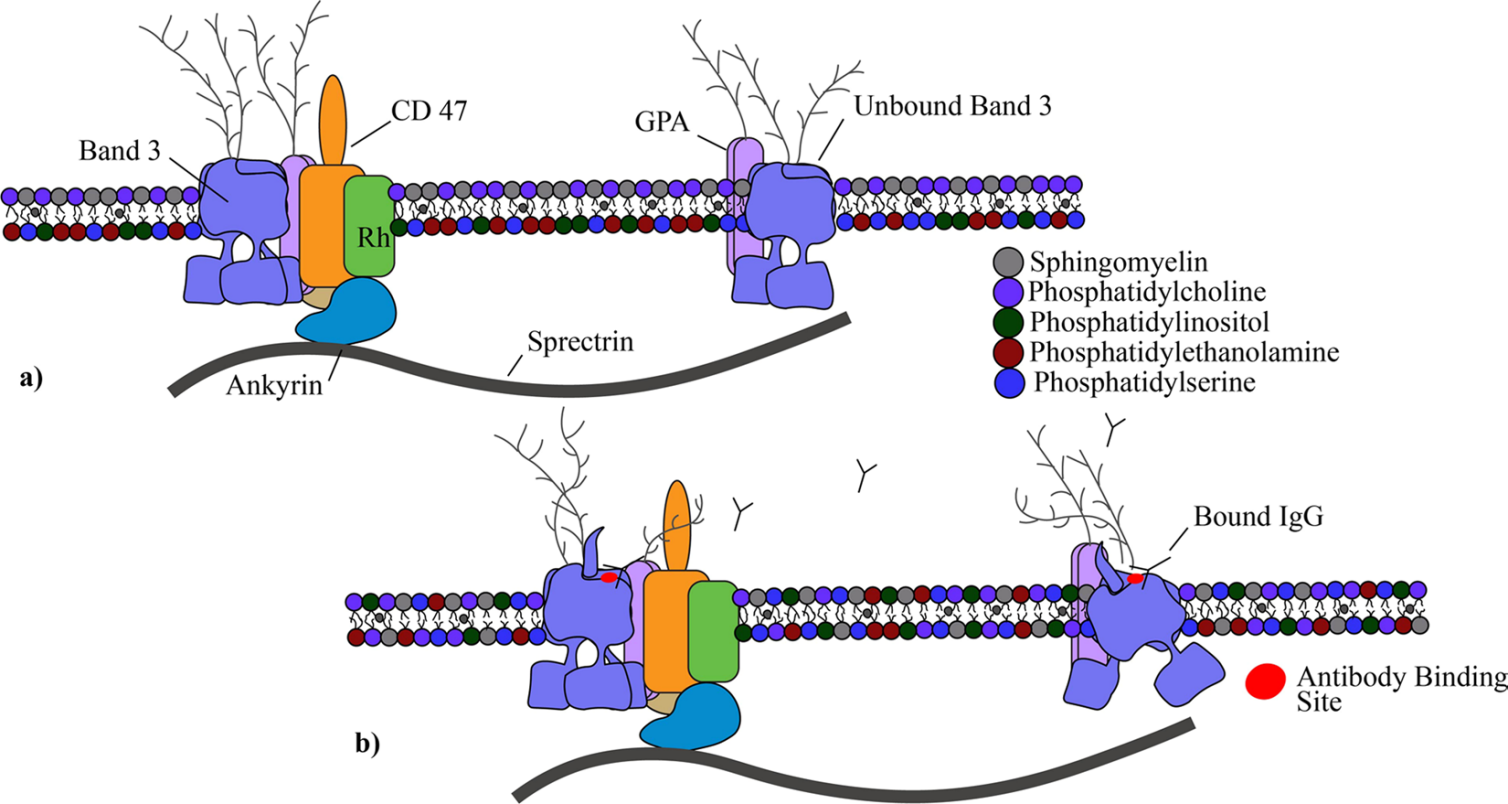
A visualization of the RBC membrane and the proposed response to shear stress by some change in Band 3 with IgG. Glycophorin A (GPA), Rhesus (Rh) protein, CD47, and ankyrin included as relevance to their inclusion in the Band 3 complex and anchoring to the RBC spectrin network. Lipids sphingomyelin, phosphatidylcholine, phosphatidylinositol, phosphatidylethanolamine and PS shown to demonstrate lipid bilayer asymmetry. (**a**) Healthy RBC membranes exhibit a continuous sorting of phospholipids to maintain consistency and peak performing proteins. The Band 3 protein exists commonly as a tetramer within a protein complex tied into the spectrin network through the protein ankyrin. Band 3 also exists as a free moving dimer. (**b**) Postulated conformational changes of Band 3 after shear exposes the senescent cell antigen and leads to binding of naturally occurring antibodies.

The importance of mechanical forces on cells to aspects of the immune system has recently been reviewed^6^. In this study, immunoglobulin binding increased with the magnitude of the stress and length of exposure (Fig. 2f,g). The results of this sublethal damage follow well-known trends for hemolysis. Findings provide evidence for a unique autoimmune response to shear forces and a new insight to RBC removal after mechanical trauma. The binding of IgG to the membrane mirrors that for senescent RBCs. Old RBCs have high levels of immunoglobulin G which serves as an opsonin, marking the cells for erythrophagocytosis by macrophages and neutrophils in the spleen^43^. The level of bound antibodies increases markedly as the cell ages. Franco found that biotinylated RBCs, though present in a low concentration, had significantly higher levels of IgG after 126 days in the human^44^. Similar experiments in the dog with biotinylated eRBCs showed a seven fold increase in bound naturally occurring antibodies (NAbs)^45^. NAbs binding to band 3 in the RBC is known and has been proven to promote phagocytosis of the cell^46^. To explain the underlying process determining the effect of aging, Low proposed that the formation and attachment of methemoglobin to the interior surface of the cell membrane promoted IgG binding via aggregation of transmembrane protein band 3^33^. We hypothesize that deformation of the cell by shear results in conformational or translational motion of band 3 to expose epitopes for NAbs and an autoimmune response similar to that from senescence. The concept is illustrated in Fig. 5.

The effects of shear on RBCs found in this research might also be compared to autoimmune hemolytic anemias, characterized by the attachment of immunoglobulin IgG and/or IgM^47^. Binding of immunoglobulins G and/or M to RBCs is the hallmark of autoimmune hemolytic anemia, “not an uncommon” clinical condition according to Chaudhary, with ultimate loss of RBCs by complement activation and the reticuloendothelial system. IgG, a relatively poor activator of the complement system^47^, leads primarily to removal of RBCs in the spleen^48^. With loss of RBCs and putative IgG binding, the cardiovascular patients groups mentioned above can be said to have a mechanically induced auto-immune hemolytic anemia.

With the prospect of an immune component to RBC removal, the relative importance of RBC deformability and IgG opsonization remains an open question. Poor RBC deformability has long been recognized for both senescent and mechanically traumatized RBCs. Working independently, Sutera and Shiga observed the flow of density separated, older cells in the rheoscope and reported increased stiffness^49,50^ and many groups have found decreased deformability after non-physiologic shear of a non-segregated population of RBCs. Stiff red blood cells are challenged by the small dimensions of the trabeculated surfaces in the spleen. Notably, perfusion of an isolated rabbit spleen *ex vivo* with a mixture of sheared (<10 N/m^2^) and unsheared RBCs in sera demonstrated selective removal of the traumatized cells^51^. Some clinical work supports the significance of altered rheology. Abridged RBC lifespans noted above for prosthetic heart valve patients correspond to reports of increased rigidity of red blood cells^52,53^. Moreover, decreasing filterability of RBCs isolated from valve patients has been correlated with lower whole blood hematocrit^53^, a result possibly due to bound proteins^54^. Another aspect of the RBC survival in the circulation is opsonization and phagocytosis in the spleen^43^. While reduced RBC deformability will slow passage through the spleen and thereby facilitate recognition of opsonins, it does not, at least *in vitro*, promote phagocytosis^32^. In studying diamide treated RBCs, Safeukui concluded splenic sequestration did not depend on reduced membrane deformability and suggested aggregation of membrane proteins such as band 3 led to splenic entrapment^29^. Thus, rheology and immunology seemingly act together to set circulatory lifespan of senescent and traumatized RBCs.

Some have suggested exposure of phosphatidylserine and apoptosis-like processes, eryptosis, as a mechanism for senescence, although this view no longer seems to be held generally^41,55^ and, in fact, PS levels are not high in aged RBCs^44^. Likewise, at 1–2%, only a very small fraction of RBCs displayed phosphatidylserine after shear (Fig. 4b), even at the longest exposure. However, the extent of PS exposed in this subpopulation did grow with exposure time (Fig. 4c). The nature of this group of RBCs is unclear, and while they are affected by shear, they do not appear to be a result of mechanical trauma since the fraction of PS positive cells did not increase with exposure time (Fig. 4b).

As RBCs age, they also lose membrane and hemoglobin by shedding vesicles or microparticles, which has been suggested as a mechanism to rid the cell of harmful species^56^. Older cells are known to be smaller. Unsheared control cells binding IgG were smaller than other cells in the sample and presumably represented a senescent subpopulation. Interestingly, those sheared cells binding IgG were also a smaller size sub-population. Thus, senescent and mechanically traumatized cells share displayed increased IgG binding, reduced deformability, smaller size and microparticle shedding.

While ventricular assist devices and prosthetic heart valves extend the lives of many patients who suffer with cardiovascular problems, they operate with regions of flow where blood experiences high shear stresses^57^ similar to conditions yielding the results of Fig. 2f. The stresses can damage cells leading to complications like anemia and thrombosis so that the discovery of increased IgG binding to RBCs after shear may have important clinical applications. Currently, a good indicator of sublethal damage is not available, though assays for bound IgG or possibly another chemical marker of senescence^38^ may meet that need. IgG should be feasible since its measurement on RBCs is already important to monitoring of patients with autoimmune hemolytic anemia^6^. Thus, it could prove to be an effective method for assessing how well patients adapt to medical devices with extracorporeal flow and for anticipating which individuals are more likely to suffer a complication like stroke, thrombosis or gastrointestingal bleeding.

An ongoing challenge is to translate flow characteristics from computer simulations into predictions of measurable blood damage. IgG binding should be valuable to engineers as a guide to further improvements in device design to reduce stress well below levels discernible by hemolysis. Linking flow history of blood to an experimental measure of the cell damage could facilitate making further improvements in design and operating conditions. In particular, assessing the level of sublethal damage to cells is essential to progress in addressing abbreviated RBC lifespans.

## Materials and Methods

### Blood collection

Venous blood was collected from healthy adult donors (n = 10), aged 18–64, in 3.2% sodium citrate vacutainers (VWR). All blood collection was done following procedures approved by the University of Oklahoma Institutional Review Board (IRB). Donors were informed of the overall goal of our research prior to donations and informed consent was obtained. Blood donations were kept confidential with no identifying documents. Experimental procedures were also performed according to approved methods set and approved by the IRB at the University of Oklahoma. All RBCs were isolated with a series of three isotonic saline solution (147.5 mM NaCl) washes, followed by re-suspension in a modified Ringer’s Solution (147.5 mM NaCl, 4 mM KCl, 2.25 mM CaCl_2_, and 10 mM glucose, with 0.05 g/L of Human Serum Albumin). Post initial centrifuge of donor blood, plasma was collected via Pasteur pipette and collected for later use. With each isotonic wash, supernatant and buffy coat were removed by Pasteur pipette. After re-suspension in modified Ringer’s solution, a Moxi Z Cell Counter (ORFLO) was used to determine RBC concentration and allowed for adjusting to an overall sample concentration of 5 × 10^9^ cells/mL (~37% hematocrit).

### Shear methods *in vitro*

Two methods were used to expose RBCs to shear in this study—a viscometer and microfluidics channels (Fig. 1). Both approaches involved shearing cells in a controlled environment for a given period of time. In each case, mechanically traumatized cells were collected for testing following shear with unsheared cells providing a control. The use of a specially designed Hercules Hi-Shear Couette viscometer with R = 1.9825 cm, G_1_ = G_2_ = 0.06 mm and G_3_ = 0.8 mm (Kaltec Industries) gave initial results. Cone-and-plate and parallel plate end regions of the bob were machined to match the shear stress in the concentric cylinder portion of the sample. Using this method the shear stress was varied while maintaining exposure time at two minutes. Shear stresses tested with the Couette viscometer were over the range of 10–130 Pa. Each test used an aliquot of 4.1 mL of the washed RBC suspension prepared as described above.

The second shear method involved the use of microfluidic channels to mimic more closely the flow conditions of high stress, short exposure times exhibited by VADs and prosthetic heart valves. The microfluidic shearing devices contained a constricted region that was designed to expose RBCs transiently (i.e. msec) to high shear conditions^58^. The cross-section of the constricted region was 45 μm wide x 60 μm tall, and the constriction length was varied from 0.7 to 11.6 mm to provide different high shear exposure times.Channels were prepared from poly-dimethyl siloxane (PDMS), using a Sylgard® 184 Silicone Elastomer kits (Dow Corning), as previously described^58^. Before running, the channel and tubing were charged with the modified Ringers solution. Flow through channels was created using an approximately 3 cm segment of tubing (1.57 mm I.D., Silastic® Laboratory) attached to a 1 mL syringe in a syringe pump. A constant shear environment on the cells is accomplished by maintaining a constant flow rate through the channels. A straight channel microfluidic device (1500 μm wide x 60 μm tall) with no constriction and low shear was used as an additional control.

### Flow cytometry analysis

Flow cytometry data was collected using a BD Accuri C6 Flow Cytometer (BD Sciences). RBC positive events were identified foremost by anti-CD235a-FITC (glycophorin A; eBioscience) binding.

### IgG binding

Immediately following shear exposure, the cells were collected and suspended to a concentration of 10^6^/mL and then incubated for 45 min at 37 °C with autologous plasma to permit autoantibody IgG binding to the cell surfaces. Afterwards, cells resuspended at a concentration of 10^6^/mL were incubated for an additional 60 min with both 5 μL (0.25 μg)/test mouse anti-CD235a-FITC (eBioscience) and 5 μL/ test mouse anti-IgG Fc Alexa Fluor 647(Biolegend) conjugates before analysis with flow cytometry or fluorescent microscopy. To account for non-specific contributions, Alexa Fluor 647 Mouse IgG2a, k Isotype Ctrl Antibody (Biolegend) was used, with no apparent binding present in control or experimental tests.

### Phosphatidylserine externalization

In addition to anti-CD235a, a second probe used was a marker for phosphatidylserine, Annexin V Alexa Fluor 647(ThermoFisher Sci). Following shear stress, cells were suspended to 10^6^ cells in 0.5 mL Ringers solution. The cellular suspension was then labeled with 5 µL of CD235a-FITC(eBioscience) and 5 µL Annexin V Alexa Fluor® 647 according to company protocol. After a 60 min. incubation period at 37 °C, cell and microparticle samples in media were then analyzed by flow cytometry.

### Confocal microscopy

Confocal microscopy images were taken with a Leica SP8 scanning confocal microscope at the Samuel Roberts Noble Microscopy Laboratory. Fresh live cells in media were imaged after labeling as described above for flow cytometry. Images were then processed using FIJI-ImageJ software to remove background noise and conduct further analysis. In each of n = 3 experiments, 50 cells were evaluated for increased fluorescence above background noise. Cells determined positive for anti-IgG Fc Alexa Fluor 647 were then further evaluated for amount of fluorescence above background by measuring the mean fluorescence.

### Statistics

In each experiment, significance was assessed using Excel statistical analysis ToolPak one-way ANOVA with further post hoc Tukey honest significant difference (HSD). The Tukey HSD tests were used to determine specifically how many of the individual tests showed significance when compared directly. Significance was defined as p < 0.05. All average values are reported as the mean ± standard error.

## Data Availability

Data sets generated for this study are available from the corresponding author on reasonable request.

## References

[1] Korenaga R (1994). Laminar flow stimulates Atp- and shear-stress dependent nitric oxide production in cultured bovine endothelial cells. Biochem Bioph Res Co.

[2] Kuchan MJ, Frangos JA (1994). Role of calcium and calmodulin in flow-induced nitric oxide production in endothelial cells. Am J Physiol.

[3] Diamond SL, Eskin SG, Mcintire LV (1989). Fluid flow stimulates tissue plasminogen activator secretion by cultured human endothelial cells. Science.

[4] Ando J, Yamamoto K (2009). Vascular mechanobiology: endothelial cell responses to fluid shear stress. Circ J.

[5] Ando J (1994). Shear-Stress inhibits adhesion of cultured mouse endothelial cells to lymphocytes by downregulating VCAM-1 expression. Am J Physiol.

[6] Huse M (2017). Mechanical forces in the immune system. Nat Rev Immunol.

[7] Poelmann RE, Groot ACGD, Hierck BP (2008). The development of the heart and microcirculation: role of shear stress. Med Biol Eng Comput.

[8] le Noble F (2004). Flow regulates arterial-venous differentiation in the chick embryo yolk sac. Development.

[9] Brown AJ (2016). Role of biomechanical forces in the natural history of coronary atherosclerosis. Nat Rev Cardiol.

[10] Tarbell JM, Shi ZD, Dunn J, Jo H (2014). Fluid mechanics, arterial disease, and gene expression. Annu Rev Fluid Mech.

[11] Bluestein D, Chandran KB, Manning KB (2010). Towards non-thrombogenic performance of blood recirculating devices. Ann Biomed Eng.

[12] Slepian MJ (2017). Shear-mediated platelet activation in the free flow: perspectives on the emerging spectrum of cell mechanobiological mechanisms mediating cardiovascular implant thrombosis. J Biomech.

[13] Carter J, Hristova K, Harasaki H, Smith WA (2003). Short exposure time sensitivity of white cells to shear stress. Asaio J.

[14] Lewis CS, Alsmadi NZ, Snyder TA, Schmidtke DW (2018). Effects of transient exposure to high shear on neutrophil rolling behavior. Cell Mol Bioeng.

[15] Jilma-Stohlawetz P (2016). Acquired von Willebrand factor deficiency caused by LVAD is ADAMTS-13 and platelet dependent. Thromb Res.

[16] Nascimbene A (2017). von Willebrand factor proteolysis by ADAMTS-13 in patients on left ventricular assist device support. J Heart Lung Transplant.

[17] Brinsfield DE, Hopf MA, Geering RB, Galletti PM (1962). Hematological changes in long-term perfusion. J Appl Physiol.

[18] Shapira Y, Vaturi M, Sagie A (2009). Hemolysis associated with prosthetic heart valves a review. Cardiol Rev.

[19] Taimeh Z (2017). Erythrocyte aging as a mechanism of anemia and a biomarker of device thrombosis in continuous-flow left ventricular assist devices. J Heart Lung Transpl.

[20] Barrett, K. E., B. M., Scott, B. & Heddwen, L. B. *Ganong’s review of medical physiology*. 25th edn, 555 (McGraw-Hill Education, 2016).

[21] Velker JA, Mcintire LV, Lynch EC (1977). Alteration of erythrocyte deformability due to shear stress as assessed by nuclepore filters. T Am Soc Art Int Org.

[22] Simmonds MJ, Atac N, Baskurt OK, Meiselman HJ, Yalcin O (2014). Erythrocyte deformability responses to intermittent and continuous subhemolytic shear stress. Biorheology.

[23] O’Rear EA, Udden MM, McIntire LV, Lynch EC (1982). Reduced erythrocyte deformability associated with calcium accumulation. Biochim Biophys Acta.

[24] Lee SS (2004). Shear induced damage of red blood cells monitored by the decrease of their deformability. Korea-Aust Rheol J.

[25] Lee SS (2007). Strain hardening of red blood cells by accumulated cyclic supraphysiological stress. Artif Organs.

[26] Watanabe N (2007). Deformability of human red blood cells exposed to a uniform shear stress as measured by a cyclically reversing shear flow generator. Physiol Meas.

[27] Nanjappa BN, Chang HK, Glomski CA (1973). Trauma of the erythrocyte membrane associated with low shear stress. Biophys J.

[28] Mitlyng BL, Chandrashekhar Y, Furne JK, Levitt MD (2006). Use of breath carbon monoxide to measure the influence of prosthetic heart valves on erythrocyte survival. American Journal of Cardiology.

[29] Safeukui I (2018). Sensing of red blood cells with decreased membrane deformability by the human spleen. Blood Adv.

[30] Kameneva MV (1995). Mechanisms of red blood cell trauma in assisted circulation. Rheologic similarities of red blood cell transformations due to natural aging and mechanical stress. Asaio J.

[31] Rubin O, Canellini G, Delobel J, Lion N, Tissot JD (2012). Red blood cell microparticles: clinical relevance. Transfus Med Hemother.

[32] Baerlocher GM, Schlappritzi E, Straub PW, Reinhart WH (1994). Erythrocyte deformability has no influence on the rate of erythrophagocytosis *in vitro* by autologous human monocytes/macrophages. Br J Haematol.

[33] Low PS, Waugh SM, Zinke K, Drenckhahn D (1985). The role of hemoglobin denaturation and band 3 clustering in red blood cell aging. Science.

[34] Schluter K, Drenckhahn D (1986). Co-clustering of denatured hemoglobin with band 3: its role in binding of autoantibodies against band 3 to abnormal and aged erythrocytes. P Natl Acad Sci USA.

[35] Hornig R, Lutz HU (2000). Band 3 protein clustering on human erythrocytes promotes binding of naturally occurring anti-band 3 and anti-spectrin antibodies. Exp Gerontol.

[36] Kay MMB (1978). Role of Physiologic Autoantibody in the removal of senescent human red cells. J Supramol Str Cell.

[37] Kay M (2005). Immunoregulation of cellular life span. Ann N Y Acad Sci.

[38] Lutz, H. U. & Bogdanova, A. Mechanisms tagging senescent red blood cells for clearance in healthy humans. *Front Physiol* **4**, doi:ARTN 38710.3389/fphys.2013.00387 (2013). 10.3389/fphys.2013.00387 PMC3872327 24399969

[39] Bosman GJCGM, Werre JM, Willekens FLA, Novotny VMJ (2008). Erythrocyte ageing *in vivo* and *in vitro*: structural aspects and implications for transfusion. Transfusion Med.

[40] Fischer TM (1980). On the energy dissipation in a tank-treading human red blood cell. Biophys J.

[41] Qadri SM, Bissinger R, Solh Z, Oldenborg PA (2017). Eryptosis in health and disease: a paradigm shift towards understanding the (patho)physiological implications of programmed cell death of erythrocytes. Blood Rev.

[42] Ghashghaeinia M (2012). The impact of erythrocyte age on eryptosis. Brit J Haematol.

[43] Meinderts SM (2017). Human and murine splenic neutrophils are potent phagocytes of IgG-opsonized red blood cells. Blood Adv.

[44] Franco RS (2013). Changes in the properties of normal human red blood cells during *in vivo* aging. Am J Hematol.

[45] Rettig MP (1999). Evaluation of biochemical changes during *in vivo* erythrocyte senescence in the dog. Blood.

[46] Simak J, Gelderman MP (2006). Cell membrane microparticles in blood and blood products: potentially pathogenic agents and diagnostic markers. Transfus Med Rev.

[47] Chaudhary RK, Das SS (2014). Autoimmune hemolytic anemia: from lab to bedside. Asian J Transfus Sci.

[48] Strobel E (2008). Hemolytic transfusion reactions. Transfus Med Hemother.

[49] Sutera SP (1985). Age related changes in deformability of human erythrocytes. Blood.

[50] Shiga T, Sekiya M, Maeda N, Kon K, Okazaki M (1985). Cell age dependent changes in deformability and calcium accumulation of human erythrocytes. Biochimica Et Biophysica Acta.

[51] Sandza JG, Clark RE, Weldon CS, Sutera SP (1974). Subhemolytic trauma of erythrocytes: recognition and sequestration by spleen as a function of shear. T Am Soc Art Int Org.

[52] Johnsson R, Harjola PT, Siltanen P (1981). Effect of pentoxifylline on red cell flexibility in arterio-sclerotic patients and in patients with heart valve prosthesis. Scand J Clin Lab Inv.

[53] Orear EA, Udden MM, Farmer JA, Mcintire LV, Lynch EC (1984). Increased intracellular calcium and decreased deformability of erythrocytes from prosthetic heart valve patients. Clin Hemorheol.

[54] Koyama T, Kikuchi Y (1982). Reduced red cell filterability due to red cell plasma protein interactions. Biorheology.

[55] Chung SM (2007). Lysophosphatidic acid induces thrombogenic activity through phosphatidylserine exposure and procoagulant microvesicle generation in human erythrocytes. Arterioscler Thromb Vasc Biol.

[56] Ratajczak MZ (2006). Microvesicles: from “dust to crown”. Blood.

[57] Quinlan NJ, Dooley PN (2007). Models of flow-induced loading on blood cells in laminar and turbulent flow, with application to cardiovascular device flow. Ann Biomed Eng.

[58] Alsmadi, N. Z. *et al*. Constricted microfluidic devices to study the effects of transient high shear exposure on platelets. *Biomicrofluidics*, **11**, doi:Artn 06410510.1063/1.4989386 (2017). 10.1063/1.4989386 PMC5705242 29204246

